# Type of medication for opioid use disorder is associated with distinct risk profiles among an urban community sample of people who use opioids

**DOI:** 10.21203/rs.3.rs-10252075/v1

**Published:** 2026-10-12

**Authors:** Grace Yi, Lauren Dayton, Kenneth Feder, Olivia Sugarman, Seun Falade-Nwulia, Mudia Uzzi, Carl Latkin

## Abstract

Background Medications for opioid use disorder (MOUD), such as methadone or buprenorphine, reduce opioid use and mortality. However, many people who use opioids (PWUO) continue to use non-prescription opiates during treatment. Little is known about how engagement in different types of MOUD used may be associated with ongoing drug use patterns, overdose history, and risk profiles. In this study, we examined whether using methadone, buprenorphine, or no MOUD was associated with varying sociodemographic characteristics, substance use behaviors, and overdose experiences among PWUO in Baltimore, Maryland. Methods We conducted a cross-sectional analysis of baseline survey data from a community-based study including N = 897 PWUO living in Baltimore. Data were collected between December 2022 and August 2024. Participants were categorized into currently receiving methadone, buprenorphine, or no MOUD. Sociodemographic characteristics, drug use patterns, and overdose history were compared using Pearson chi-square tests of association. Multivariable logistic regression models, adjusted for immutable sociodemographic traits, were used to examine associations between MOUD modality and drug-related outcomes. Results MOUD modality differed significantly by race and sex. Those receiving buprenorphine were more frequently Black and less frequently female than those receiving methadone. After adjustment for sociodemographic factors, individuals on methadone had higher odds of weekly injection drug use than those receiving no MOUD (adjusted odds ratio (aOR) 1.59, 95% CI: 1.09–2.34), and lower odds of overdose within the previous six months (aOR 0.62, 95% CI 0.42–0.92). Those on buprenorphine had lower odds of weekly stimulant and opioid co-use than those receiving no MOUD (aOR 0.56, 95% CI: 0.36–0.85), but greater odds of having experienced multiple lifetime overdoses (aOR 1.58, 95% CI: 1.07–2.34). Frequent fentanyl use (weekly or more) was high across all groups. Conclusions PWUO receiving differing MOUD modalities had distinct sociodemographic characteristics, drug use behaviors, and overdose histories. These findings suggest that MOUD modality may reflect different treatment trajectories and risk profiles. Harm reduction and treatment interventions should be tailored to the specific needs of individuals receiving different forms of MOUD, including sustained injection-related harm reduction services within treatments programs and enhanced overdose prevention strategies for people receiving buprenorphine.

